# A Rare Tumor with a Very Rare Initial Presentation: Thymic Carcinoma as Bone Marrow Metastasis

**DOI:** 10.1155/2017/6497376

**Published:** 2017-01-02

**Authors:** Sonam Sharma, Leelavathi Dawson

**Affiliations:** Department of Pathology, Vardhman Mahavir Medical College & Safdarjung Hospital, New Delhi, India

## Abstract

Tumors of thymus gland are rare and account for 0.2% to 1.5% of all the neoplasms. They constitute a heterogeneous group that has an unknown etiology and a complex as well as varied biology. This has led to difficulty in their histological classification and in predicting their prognostic and survival markers. Among them, thymic carcinoma is the most aggressive thymic epithelial tumor exhibiting cytological malignant features and a diversity of clinicopathological characteristics that can cause diagnostic dilemmas, misdiagnosis, and therapeutic challenge. We herein describe a case of a 60-year-old man who while undergoing evaluation for the cause of pancytopenia was discovered having bone marrow metastasis from an asymptomatic thymic carcinoma. Bone marrow metastasis is an extremely rare initial presentation of thymic carcinoma with only few cases reported in the literature.

## 1. Introduction

Thymic tumors are relatively rare, with their incidence being 0.13 per 100,000 person-years [1]. They comprise spectrum of neoplasms arising from or differentiating towards thymic cellular constituents, including thymic epithelial (thymomas, thymic carcinomas, and neuroendocrine tumors), germ cell, lymphoid/haematopoietic, and mesenchymal tumors. Among them, thymic carcinomas are the most aggressive variant that represent approximately 0.06% of all malignancies and 7–25% of all thymic neoplasms [2, 3]. They correspond to type C thymoma category according to the early World Health Organization (WHO) classification of thymic epithelial tumors [4]; however this category has been excluded from the current WHO classification of tumors of the thymus [5]. They differ from thymomas clinically, by being asymptomatic in 30% cases, in having more propensity for loco-regional invasion of surrounding mediastinal structures with metastasis and histologically by the presence of overt malignant cytologic features [6, 7]. Extrathoracic metastasis due to metastasis of thymic carcinoma is a very rare event to occur, the exact incidence of which is yet unknown [8, 9]. Various sites like liver, lungs, lymph nodes, kidney, brain, and adrenal glands have been documented in the literature for extrathoracic metastasis [9–11]. Bone marrow metastasis is usually seen in the late stages of thymic tumors [12]. An initial clinical presentation with involvement of bone marrow by this aggressive tumor prior to or at the time of its diagnosis is extremely rare [13, 14]. Paucity of the knowledge of this malignancy due to its low incidence along with the lack of understanding about its prognostic markers, diagnostic approach, and treatment prompted us to present this case. The present case study describes a patient with bone marrow metastasis as the first evidence of occult thymic carcinoma, with emphasis on the role of various immunohistochemical markers. This case also enlightens the readers to be aware about initial bone marrow metastasis by this rare tumor.

## 2. Case Presentation

A 60-year-old man, shopkeeper by occupation, presented with history of fever for the last two months. The fever was low grade, intermittent, without any diurnal variation, not associated with chills or rigors, night sweats, headache, rashes, and weight loss. He was a smoker for the last 10 years, nonalcoholic, and vegetarian by diet. His past medical and surgical history was unremarkable. Only medication that he took on and off was an antipyretic for fever. His travel history was noncontributory. He also denied any personal and family history of any major illness. His general physical examination revealed pallor while his systemic examination was normal. Abnormal laboratory parameters were dimorphic anemia with hemoglobin level of 8.5 gm/dl, a peripheral blood leukocyte count of 2500/*µ*l, and a platelet count of 40,000/*µ*l, revealing pancytopenia. The erythrocyte sedimentation rate was 15 cumm/hr. Urine and blood cultures were negative. Mantoux's test was negative. Serological tests for human immunodeficiency virus and hepatitis virus were nonreactive. Liver and kidney function tests were within normal limits.

**Figure 1:**
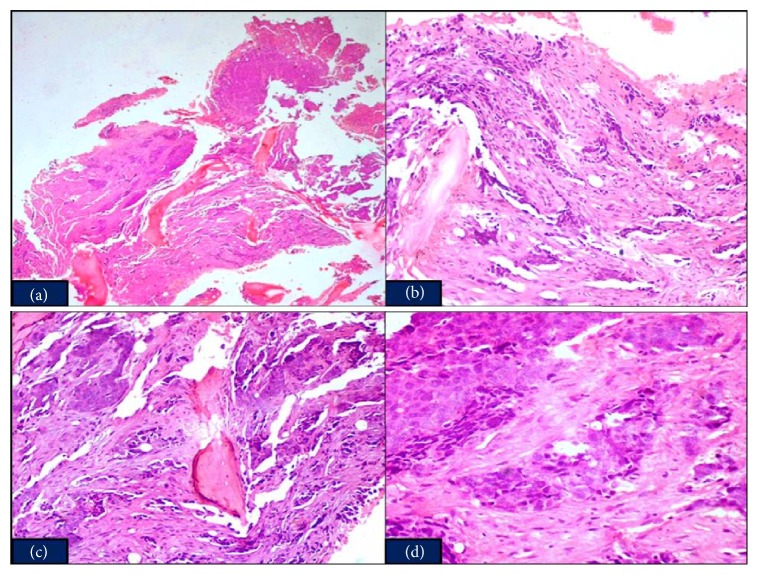
(a) Bone marrow biopsy (H&E ×4). (b) Cords and sheets of malignant tumor cells with surrounding desmoplastic reaction (H&E ×100). (c) Tumor cells intermingled with normal haematopoietic cells of the bone marrow (H&E ×200). (d) Poorly differentiated tumor cells (H&E ×400).

A posterior superior iliac spine bone marrow biopsy was done to detect the cause of pancytopenia. Histopathological sections of the trephine biopsy showed an extensive involvement of bone marrow by sheets, clusters, and cords of poorly differentiated tumor cells with indistinct cell borders, high nuclear to cytoplasmic ratio, moderate amount of eosinophilic cytoplasm, round to oval vesicular nucleus, indistinct nucleoli, and high mitosis. At places, desmoplastic reaction was seen (Figures 1(a), 1(b), 1(c), and 1(d)). Based on these features, a diagnosis of a metastatic malignant tumor was made.

**Figure 2:**
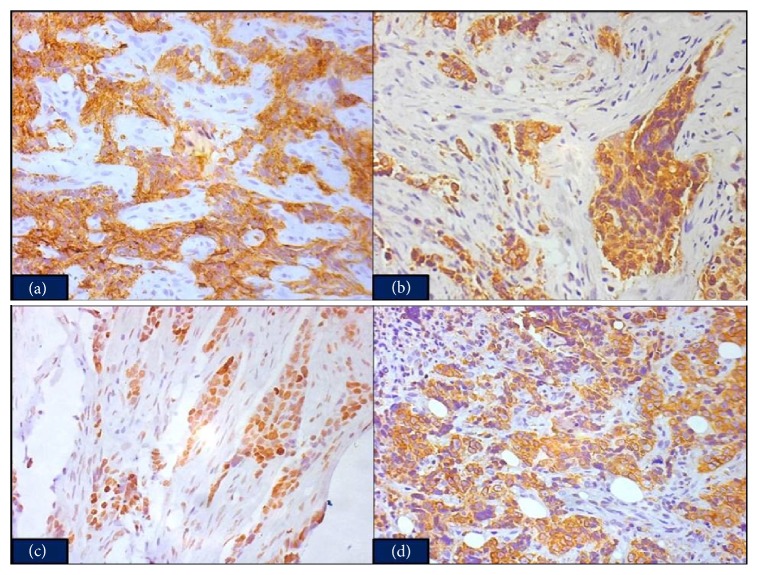
(a) Immunohistochemical staining for CD5 shows strong, diffuse cytoplasmic staining of the tumor cells (×200). (b) Bcl-2 cytoplasmic immunopositivity of the tumor cells (×200). (c) Immunohistochemical staining for p53 shows strong nuclear staining of the tumor cells (×200). (d) Tumor cells exhibiting positive CD117 expression (×200).

A panel of immunohistochemical markers were put for tumor characterization. Tumor cells exhibited strong positive expression for pan cytokeratin (CK) [AE1 and AE3], epithelial membrane antigen (EMA) [E29], CD5 [4C7], Bcl-2 [100/D5], p53 [D07], CD117 [YR145], and p63 [4A4] (Figures 2(a), 2(b), 2(c), and 2(d)) and negative expression for leukocyte common antigen (LCA) [PD7/26/16 and 2B11], CD3 [PS1], CD1a [O10], CD99 [HO36-1.1], CD20 [L26], CD30 [CON6D/B5], CD15 [BRA4F1], thyroid transcription factor (TTF-1) [8G7G3/1], Wilm's tumor suppressor gene 1 protein (WT-1) [CAN-R9(IHC)-56-2], placental alkaline phosphatase (PLAP) [PL8/F6], CK7 [OV-TL 12/30], CK20 [EPR1622Y], estrogen receptor (ER) [SP1], progesterone receptor (PR) [SP2], Muc-1 [EPR1023], synaptophysin [Snp88], chromogranin [LK2H10], CD34 [QBend/10], vimentin [V-9], desmin [33], S100 [15E2E2], prostate specific antigen (PSA) [ErPr8], antimelanoma [HMB-45], and terminal deoxynucleotidyl transferase (TdT) [polyclonal].

**Figure 3:**
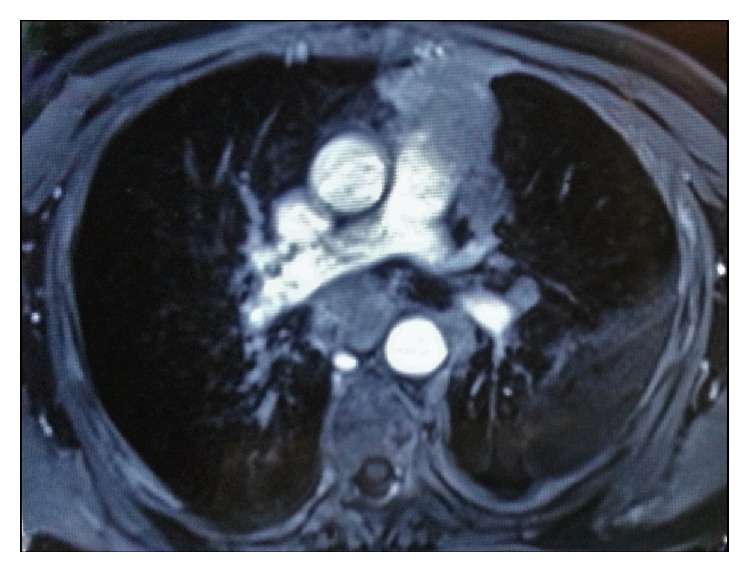
MRI chest revealing an anterior mediastinal mass which is infiltrating the surrounding lung parenchyma.

Considering the morphological and immunohistochemical findings, a diagnosis of poorly differentiated thymic epithelial tumor metastasis to the bone marrow was made. Following this, a detailed clinical and radiological workup was advised to find the primary. Computed tomography (CT) scan and Magnetic Resonance Imaging (MRI) of the chest revealed an ill-defined, approximately 58 × 45 × 38 mm in size heterogenously enhancing mass in the anterior mediastinum (Figure 3).

CT-guided core biopsy of this mediastinal mass showed histopathological and immunohistochemical features of poorly differentiated thymic carcinoma with tumor cells showing CK, EMA, CD5, p53, CD117, p63, and Bcl-2 positivity. Based on these findings, a final diagnosis of clinically occult poorly differentiated thymic carcinoma metastatic to the bone marrow was made. The tumor at initial diagnosis presented in stage IVb according to the Masaoka staging system. The patient underwent surgical excision followed by postoperative radiotherapy and four cycles of cisplatin, doxorubicin, vincristine, and cyclophosphamide (ADOC) chemotherapy. However, he succumbed to the disease eight months after the diagnosis.

## 3. Discussion

Thymic neoplasms comprise a broad category of rare lesions with a wide spectrum of clinicopathological characteristics which in turn are dependent upon their histology, tumor stage, and the existence of paraneoplastic syndromes. Their rarity also makes their recognition difficult and challenging; therefore they require a high index of suspicion and interdisciplinary management approach especially in cases when rare metastatic sites and atypical symptoms represent early symptoms of an underlying/occult malignant disease. Although the etiology and risk factors for thymic tumors are unknown, previous irradiation, genetics, and Epstein-Barr virus infections have been thought to play a role [1, 15].

Among them, thymic carcinoma is an aggressive epithelial tumor of thymic origin which is associated with a high degree of histological anaplasia, obvious cell atypia, high proliferative activity, and tendency for extensive metastasis (intra/extrathoracic) as compared to thymoma [16, 17]. Most of them arise de novo, but some arise through transformation from thymomas. It has been classified into two categories, that is, low and high grade. Low grade includes basaloid, mucoepidermoid, and well-differentiated squamous cell types and has been linked to a better chance of recovery. High grade includes anaplastic, clear cell, poorly differentiated squamous cell, sarcomatoid, and small cell/neuroendocrine types which show poor response to the treatment and have less chances of survival [17]. It commonly occurs in the 5th decade of life and predominantly in males. The patients can present with symptoms depending upon its invasiveness or metastasis like persistent cough, shortness of breath, pain or pressure in the chest, muscle weakness, difficulty in swallowing, anemia, frequent infections, back pain, and so forth; however, 30% patients can be asymptomatic [6]. Its association with paraneoplastic syndromes (myasthenia gravis, pure red cell aplasia, hypercalcemia, etc.) is extremely rare and is usually seen in cases that evolve from a preexisting thymoma [18, 19].

In the present case, the patient presented with fever only and did not have any respiratory symptoms related to the primary thymic carcinoma. He was rather diagnosed later with primary thymic carcinoma depending upon the histological and immunohistochemical findings obtained from the bone marrow biopsy submitted for detecting the cause of pancytopenia. According to the Masaoka staging system [20], the present case had advanced disease with haematogenous spread to the bone marrow (stage IVb). The scarcity of the tumor cases as well as the lack of the knowledge of bone marrow metastasis prevalence prior to the onset of the typical symptoms, during the clinical course or later in the advanced or recurrence stage, makes bone marrow metastasis a controversial clinical prognostic indicator of this tumor. However, few authors have mentioned bone marrow involvement as thymic carcinoma's initial clinical presentation like in the present case [13, 14], and others have identified it during the progression or late stages of the disease [21] while few speculated it to occur during the tumor recurrence after surgical intervention [22]. Therefore, we postulate that bone marrow involvement can occur at any stage of this aggressive tumor and thymic carcinoma should be kept in the mind while dealing with any case presenting initially as bone marrow metastasis especially in cases of unknown primary.

The management of such cases is through a multidisciplinary approach because it is important to differentiate between the thymic malignancies and the other tumors prior to initiation of the therapy as it greatly affects the prognosis and chances of survival in a patient. The present case was poorly differentiated thymic carcinoma and is classified as high-grade histological type according to the classification of Suster and Rosai [17]. This histopathological diagnosis was first made based upon the findings of bone marrow biopsy, in which the immunohistochemistry (IHC) played a pivotal role in recognition of tumor cells of thymic origin as well as in differentiating them from other tumors demonstrating similar morphology. Among them CD5, CD117, Bcl-2, p53, and p63 were the cardinal ones which helped in clinching the diagnosis as these markers, especially CD5, CD117, according to the existing literature are usually seen in thymic carcinoma [13, 23–26]. However, radiological evaluation followed by histopathology including IHC is mandatory for a firm diagnosis of primary thymic carcinoma and these diagnostic modalities also aid in differential diagnosis. In the present case, CT scan as well as MRI was quite helpful in locating the tumor in the anterior mediastinum. The main important differentials are thymoma, lung/oesophageal/thyroid carcinoma, and other carcinomas showing squamous cell carcinoma on histology, NUT carcinoma, metastatic carcinoma from breast, lung, prostate, thyroid, kidney, and other less common ones are thymic carcinoid, germ cell tumors, lymphomas, sarcomas, and melanomas.

The gold standard for the management of all thymic neoplasms regardless of stage remains complete surgical resection [27]. In cases of unresectable tumors or metastasis, neoadjuvant cisplatin based chemotherapy or radiotherapy has been recommended by some researchers [3, 28]. However, a standard effective treatment in such cases has not been well established and is still under trial [3, 13]. Nevertheless, in the era of targeted therapy, there have been investigations into specific targets in thymic neoplasms which may improve response to therapy such as epidermal growth factor receptor (EGFR) mutations, c-kit mutations, and insulin-like growth factor-1R expression [29, 30]. The prognosis of thymic carcinoma is generally poor and the tumor stage is the most important prognostic indicator [31]. In the present case, even after surgical excision, ADOC chemotherapy, and radiation, the disease progressed and the patient died. This occurrence is further supported by some authors who have documented that the patients with hematogenous metastasis at the time of the initial diagnosis have a significantly poorer prognosis than those with lymphogenous metastasis [32]. However, further large number of studies on thymic carcinoma are required to understand its complex biology and to treat this rare entity through multimodality approach.
